# A High-Sensitive Pressure Sensor Using a Single-Mode Fiber Embedded Microbubble with Thin Film Characteristics

**DOI:** 10.3390/s17061192

**Published:** 2017-05-23

**Authors:** Guanjun Wang, Xinglin Liu, Zhiguo Gui, Yongquan An, Jinyu Gu, Meiqin Zhang, Lu Yan, Gao Wang, Zhibin Wang

**Affiliations:** 1 Key Laboratory of Optoelectronic Devices and Systems of Ministry of Education and Guangdong Province, College of Optoelectronic Engineering, Shenzhen University, Shenzhen 518060, China; wangguanjun@nuc.edu.cn; 2 School of Information and Communication Engineering, North University of China, Taiyuan 030051, China; S1505033@st.nuc.edu.cn (X.L.); guizhiguo@nuc.edu.cn (Z.G.); S1505032@st.nuc.edu.cn (J.G.); S1505016@st.nuc.edu.cn (M.Z.); S1505018@st.nuc.edu.cn (L.Y.); wanggao@nuc.edu.cn (G.W.); wangzhibin@nuc.edu.cn (Z.W.); 3 Engineering Technology Research Center of Shanxi Province for Opto-Electronic Information and Instrument, Taiyuan 030051, China

**Keywords:** pressure sensing, Fabry–Perot interference, microbubble, thin film layer

## Abstract

A new fiber pressure sensor is proposed and analyzed in this paper. A commercial arc fusion splicer and pressure-assisted arc discharge technology are used here to fabricate a silica hollow microbubble from a common glass tube with the characteristics of a thin film. Then the single mode fiber is embedded into the microbubble to form a fiber Fabry–Perot interferometer by measuring the reflected interference spectrum from the fiber tip and microbubble end. As the wall thickness of the micro-bubble can reach up to several micrometers, it can then be used for measuring the outer pressure with high sensitivity. The fabrication method has the merits of being simple, low in cost, and is easy to control. Experimental results show that its pressure sensitivity can reach 164.56 pm/kPa and the temperature sensitivity can reach 4 pm/°C. Therefore, it also has the advantage of being insensitive to temperature fluctuation.

## 1. Introduction

Fiber Fabry–Perot (F-P) interferometer sensors have the advantages of ultra-compact size, high sensitivity, good reusability, and immunity to electromagnetic interference, etc. [1,2,3]. They have been widely used in many fields, such as biological medicine and down-hole oil/gas exploration [4,5,6]. They play important roles in a many sensing applications, such as measuring stress [7,8], pressure [9,10,11], temperature [12,13], and refractive index [14,15] parameters.

Based on the technology of arc discharge, chemical etching, femtosecond lasers, and other methods, many microbubble or micro-cavity structures can be formed in the fiber. The fiber Fabry–Perot interferometer is realized by measuring the reflected interference spectrum between the end of the fiber and the microbubble/microcavity. As the thickness of the bubble/cavity could be drawn or removed to the scale of several micrometers, such F-P sensors have a higher pressure sensitivity characteristic than traditional F-P interferometer sensors.

Many fabrication methods for fabricating such types of fiber pressure sensors have been reported [7,15]. Jiang once fabricated a micro-cavity by etching a multimode fiber and then fusing the fiber ends together to form the F-P interferometer. The reported fiber sensor has a pressure sensitivity of ~3.64 nm/N and a temperature sensitivity of ~2 pm/°C [7]. However, it seems the roughness of the etched concave hole was obviously influenced by the chemical etching and the sensitivity was influenced, as well. Ma fabricated a fiber microbubble at the fiber-tip by splicing a silica capillary to a single mode fiber and then melted the capillary to form an internal air cavity. Its pressure sensitivity was of ~1.37 nm/N and its temperature sensitivity was of ~2.1 pm/°C [8]. However, the thickness of the fabricated microbubble was only 6–12 μm, so its pressure sensitivity was restricted. Ma also improved the microbubble fabrication method by using the fusion splicer and pressurizing gas chamber. Then, the new fiber-tip microcavity-shaped pressure sensor was fabricated and a pressure sensitivity of ~315 pm/MPa was demonstrated [9]. Liao also demonstrated a sub-micron silica diaphragm-based fiber-tip Fabry–Perot interferometer for pressure measurement by using an improved electrical arc discharge technique. Its pressure sensitivity reached up to 1036 pm/MPa and the temperature sensitivity was only about 960 Pa/°C [10]. Wang also demonstrated a fiber pressure sensor by forming a micro air bubble at the end a single mode fiber. The cavity length was compressed under high environmental pressure. The corresponding pressure sensitivity was better than 1000 nm/kPa [11]. Ma also built a miniature fiber-tip pressure sensor by using an extremely thin graphene film as the reflection diaphragm. The graphene-based fiber sensor revealed a high-pressure sensitivity over 39.4 nm/KP when the graphene diaphragm diameter was of 25 μm [12]. Additionally, femtosecond laser micromachining technology was also utilized to fabricate the interference cavity under the single mode fiber. Liao once demonstrated a refractive index sensor based on F-P interferometry with a sensitivity of ~994 nm/RIU by using a femtosecond laser [14]. Furthermore, Wu also fabricated a fiber-optic pressure sensor by using planar photonic crystal diaphragms as the optical resonators. Its spectral shift sensitivity to pressure was up to 8.6 nm/kPa [16].

Many methods for fabricating fiber F-P interferometer sensors have been proposed. The chemical etching method has the advantage of low cost, but the disadvantage of poor spectrum characteristics and low sensitivity. The graphene film-based pressure sensor has the highest sensitivity, but the cost is not cheap, while the arc discharge-based method has the merits of low cost and relatively high sensitivity. Therefore, how we can improve the current sensitivity of the microbubble-based F-P pressure sensor is of great importance for practical applications.

In this paper, a new fabrication method of a fiber-tip microbubble-based pressure sensor is presented by utilizing the pressure-assisted arc discharge technique. The wall thickness of the micro-bubble can reach 2 μm and the pressure sensitivity can reach 164.56 pm/kPa, which is proved in our experiment. Compared with the above methods, the fabrication approach is relatively simple, highly efficient, and easy to handle. Only a fusion splicer and pressure pump were used here. Compared with the fiber-tip micro-cavity pressure sensor in [9], the microbubble sensor shows a high-pressure sensitivity due to the silica wall thickness *t* = 2 μm. It also has the advantage of being insensitive to temperature fluctuations as its temperature sensitivity was only 4 pm/°C. Additionally, as the all-fused silica spherical structure enhanced the mechanic strength and stability, this fiber-tip micro-bubble structure was very suitable for high-pressure measurement in harsh environments.

## 2. Fabrication and Principle

**Figure 1:**
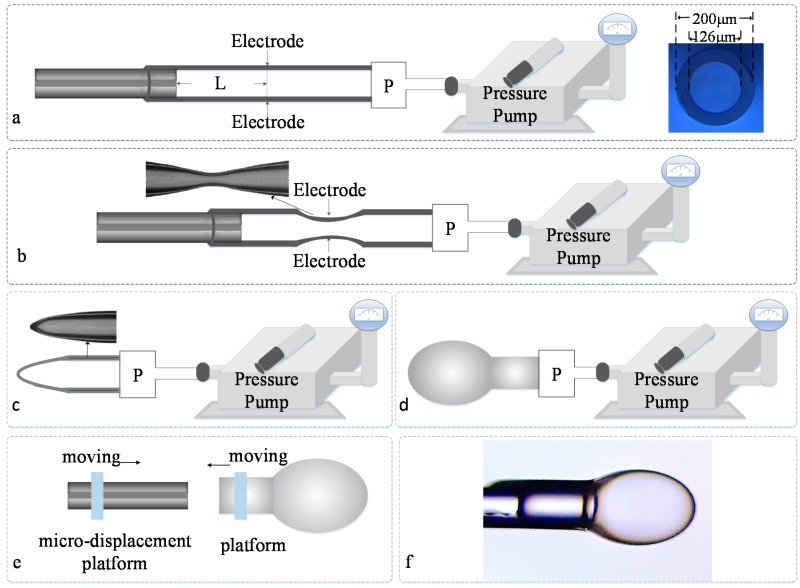
Fabrication process of the fiber pressure sensor. (**a**) Splice a glass tube to a single-mode fiber; (**b**) The diameter and thickness of the glass tube become thinner by tuning the parameters of fusion and filling pressure; (**c**) Heat and melt the glass tube to form a single-ended hollow conical structure; (**d**) A sketch showing the fiber-tip microbubble; (**e**) The single-mode fiber and microbubble were sealed together; (**f**) A sketch showing the final fiber-tip micro-bubble sensor.

In this paper, an ultrathin microbubble structure is fabricated by utilizing the pressure-assisted arc discharge technique. A glass tube with an outer diameter *D* of 200 µm, and an inner diameter *d* of 126 µm, is used here to form the microbubble. In addition, a single-mode fiber (corning company) is utilized for embedding into the microbubble to form the Fabry–Perot interference. The fabrication process of the fiber pressure sensor is shown in Figure 1. Figure 1a shows the microscope image of the capillary.

**Figure 2:**
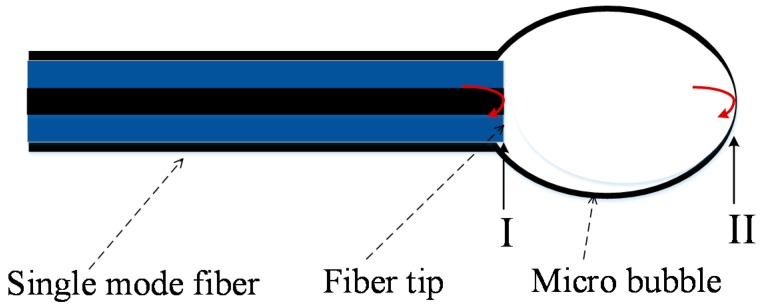
Schematic diagram of the fiber-tip F-P interferometer.

The fabrication process can be described as follows: Firstly, as was depicted in Figure 1a,b, the single-mode fiber was inserted into the glass tube and then fused by a commercial fusion splicer (Fujikura FSM 60S, Fijikura, Tokyo, Japan). The other end of the glass tube was connected to a pressure pump. Then the glass capillary region was tapered by the same fusion splicer under the condition of filling the capillary with gas at ~120 kPa from the other end that was connected with the pressure pump. By tuning the parameters of fusion and filling pressure, the diameter and thickness of the silica capillary could be thinner. Then, when discharged again, a single-ended hollow conical structure appeared, shown in Figure 1c. When altering the filling pressure and the discharge parameters of the fusion splicer, and discharged again under the condition of filling the capillary with high pressure, the hollow conical structure expanded into a microbubble structure. A uniform and smooth microbubble structure with a wall thickness of a few micrometers was realized after discharging several times, as shown in Figure 1d, while the microbubble was cut from the capillary by using a common fiber cutter. For forming the FP interference, another single-mode fiber was inserted into the micro-bubble under the microscope. The distance of the fiber end and microbubble tip could be tuned by monitoring the interference spectrum from a fiber interrogator. Finally, the single-mode fiber and microbubble were sealed together by an arc discharge for forming the pressure sensor. The sealed pressure is shown in Figure 1e. Figure 1f shows the image of the final fiber-tip micro-bubble sensor. It seems that the fiber microbubble could be tuned to be very thin by using suitable fusion and filling pressure parameters. Figure 2 depicts the schematic diagram of this fiber-tip F-P interferometer. As the wall of the fiber-embedded microbubble was uniform, smooth, and ultrathin, the end surface of the microbubble structure will have a good reflection effect, which could reflect the light back into the single mode fiber. An F-P interferometer was formed between the end of fiber and the bubble.

When subjecting the fabricated pressure sensor to different pressures, the position of the bubble end will change, while the laser beam will be reflected from the fiber end (I) and the bubble end (II). The two reflected beams then propagate back through the same fiber and generate the interference fringes, which are caused by a phase delay. A fiber interrogator can detect the reflected light. The output light intensity of the sensor is expressed as:

(1) $$I=I_{1}+I_{2}+2\sqrt{I_{1}I_{2}}\cos\gamma$$

(2) γ=4πnairdλ.

Here $I_{1}$ and $I_{2}$ are the light intensity of the two reflected waves, and γ is the difference of the phase between the two reflected beams. The relationship between the spacing of the wavelength Δλ and the change of the phase Δγ can be expressed as:

(3) Δγ=4πnairdΔλλ2.

When the cavity length *d* of the pressure sensor changes, the measured F-P interference spectrum will change correspondingly. If the difference of phase is 2π, the spacing of the wavelength can be expressed as:

(4) Δλ=λ22naird.

The pressure sensitivity is:

(5) $$K_{P}=\frac{\Delta \lambda }{\lambda }/P\;$$

(6) $$K_{P}=\frac{\lambda }{2n_{air}d}/P=\lambda /2n_{air}dP.$$

Here λ is the beam wavelength, $n_{air}$ is the refractive index of air $\left(n_{air}\sim 1\right)$, and d is the cavity length between the fiber end and microbubble tip.

## 3. Experiment and Analysis

**Figure 3:**
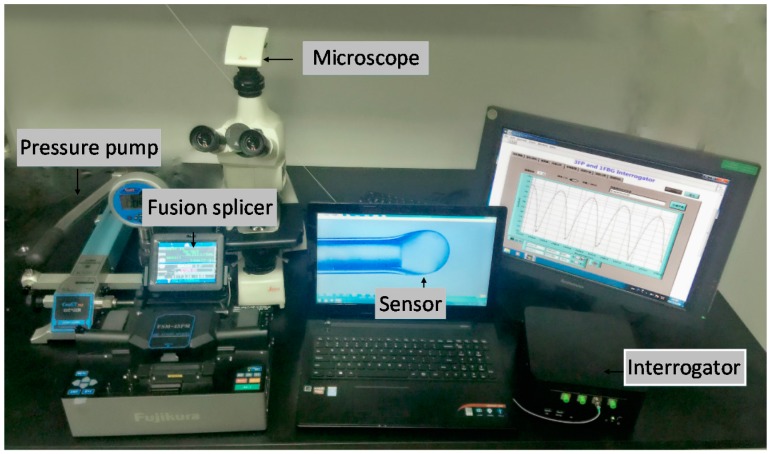
Pressure measurement system of the fiber-tip micro-bubble sensor.

The fabricated microbubble has the advantages of being ultra-thin, highly sensitive to outer pressure, being low in cost, and having good stability. It is also easy to control the length of the F-P cavity. In order to analyze the characteristics of the pressure sensor under different filling pressures, a fiber F-P interference test system was set up, which is shown in Figure 3. The fiber F-P interference testing system was composed of a fusion splicer (Fijikura FSM 60S, Fijikura, Tokyo, Japan), a pressure pump (ConST162, ConST, Beijing, China), a microscope (LEICA DM750M, LEICA, Shanghai, China), and an interrogator.

In conclusion, the fabrication process of the fiber pressure sensor could be divided into two parts: In part one, the microbubble structure was fabricated at the end of the glass tube; and in part two, the single-mode fiber was embedded into the microbubble structure under the assistance of the microscope. In this way, the proposed pressure sensor would take effect by measuring the interference spectrum of the reflected beam from the fiber tip and bubble end. Compared with previously-reported sensors, the proposed fiber-tip microbubble sensor has several advantages. As shown in Figure 1e, the wall thickness of the microbubble was ultra-thin and the cavity length of the sensor can be controlled.

**Figure 4:**
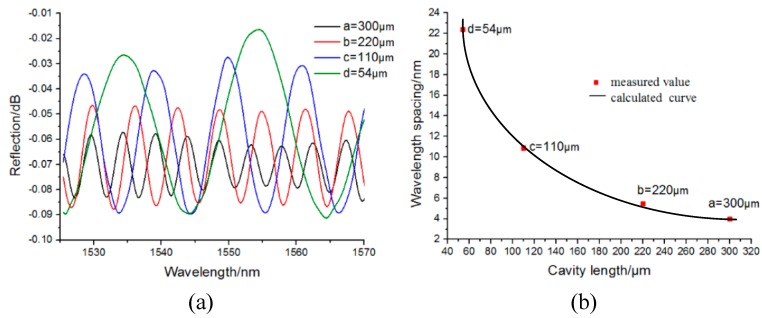
Fabry–Perot interference of the proposed pressure sensor. (**a**) Reflection spectra under different cavity lengths. (**b**) The relationship between the wavelength spacing and cavity length.

Figure 4a shows the reflection spectra of the proposed pressure sensor under different cavity lengths. Here a, b, c, and d represent the cavity lengths, which were 300 μm, 220 μm, 110 μm, and 54 μm, respectively. Figure 4b shows the fitted curve of the wavelength spacing corresponding to the different cavity lengths. From Figure 4b, the wavelength spacing Δ*λ* could be estimated as 4 nm, 5.5 nm, 10.9 nm, and 22.4 nm, respectively. Then the theoretical values of the wavelength spacing were calculated by using Equation (4). As the cavity lengths have been measured as 300 μm, 220 μm, 110 μm, and 54 μm, respectively, the corresponding wavelength spacing can be calculated to be 4 nm, 5.46 nm, 11 nm, and 22.24 nm, respectively, which agrees well with the upper measured values of the wavelength spacing.

**Figure 5:**
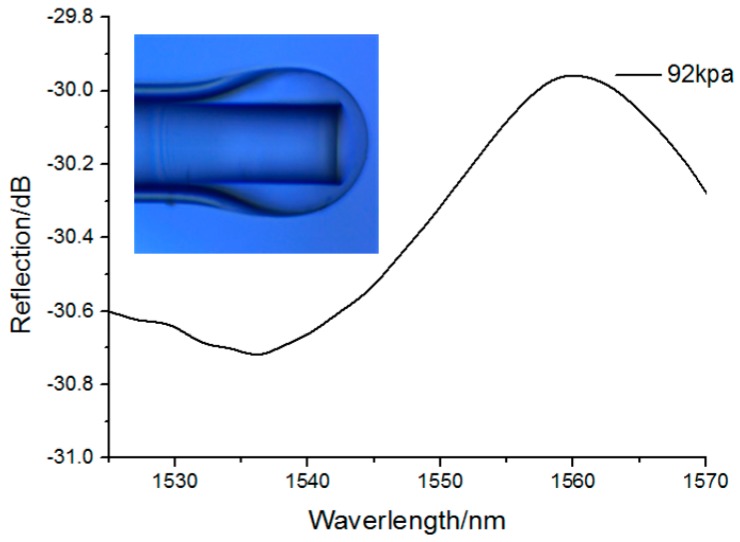
FP reflectance spectra of the proposed pressure sensor under a filling pressure of 92 kPa.

**Figure 6:**
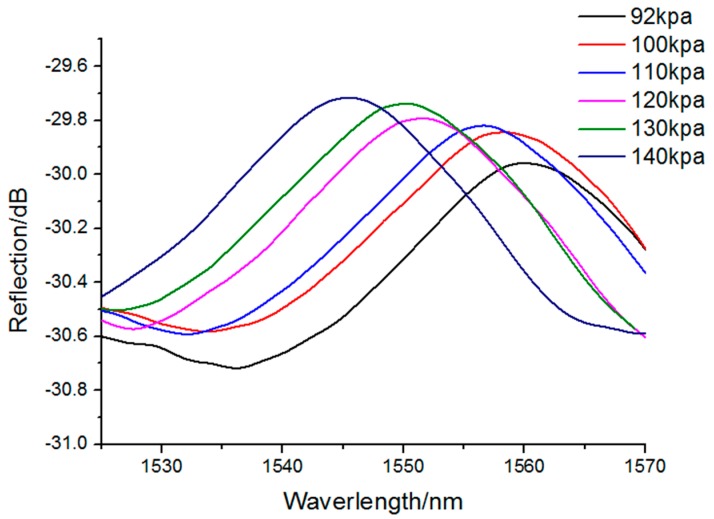
Reflection spectra of fabricated sensor under different pressures.

In the following sections, the relationship of the reflection spectrum and the outer pressure value is analyzed. Figure 5 depicts the reflection spectrum of the proposed fiber pressure sensor under an absolute filling pressure of 92 kPa. As shown in Figure 5, the spectral bandwidth was 45 nm, and the ordinate was the reflection value of the F-P cavity. For measuring the sensitivity characteristic of the proposed pressure sensor, the microbubble was put into the air chamber of the pressure pump. Tuning the pressure value of the pump, the position of the bubble end will move, and the reflection spectrum peak will shift correspondingly. Figure 6 depicts the shift of the reflection spectrum under different pressure values. Here, the cavity length *d* was set as 25 μm and the silica wall thickness of bubble end was about 2 μm. The pressure value is increased from 92 to 140 kPa. From Figure 6, it can be determined that the peak of the reflection spectrum shifts toward to the left with the increase in pressure.

**Figure 7:**
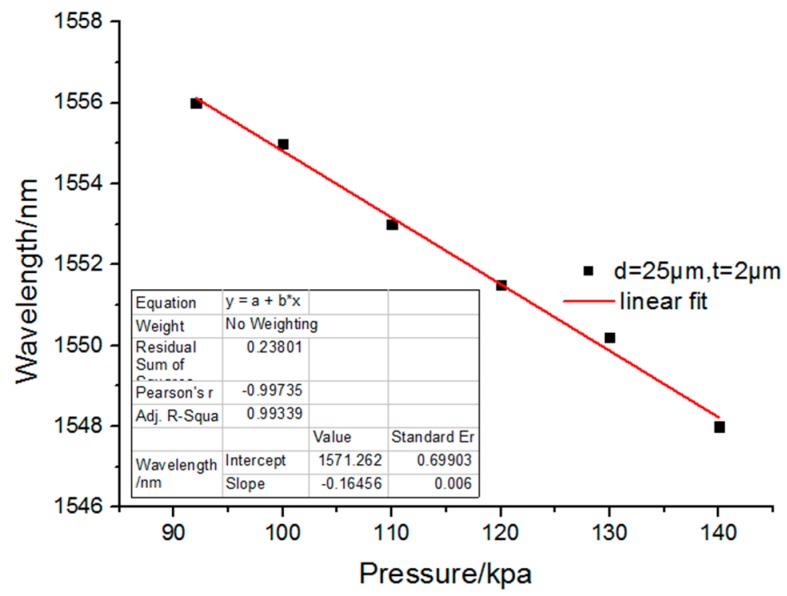
Pressure sensitivity characteristics of the sensor.

The characteristics of the microbubble structure were that the end of the microbubble deforms under the radial force. The radial force was formed when the external pressure affected the maximum radial region of the microbubbles. With the increase of pressure, the deformation of the bubble end will change obviously. According to the calculated result, the pressure sensitivity of the pressure sensor was as high as 164.56 pm/kPa, which is depicted in Figure 7.

**Figure 8:**
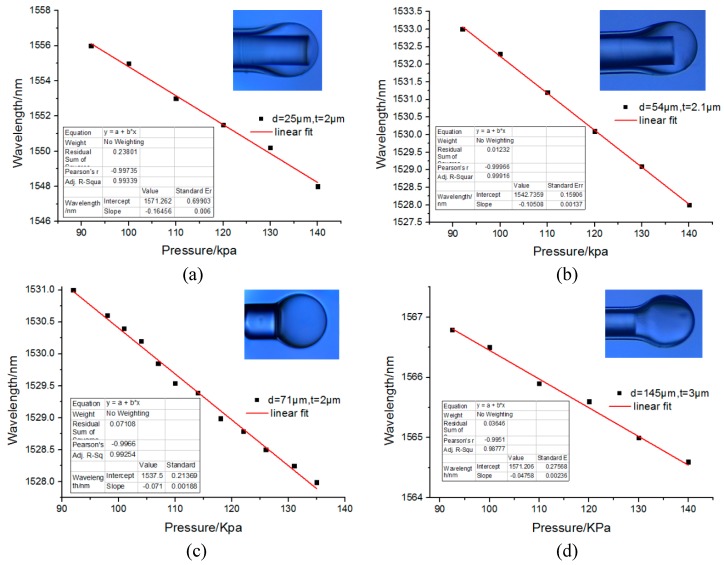
The pressure sensitivity of the microbubble under different cavity lengths: (**a**) *d* = 25 μm; *t* = 2 μm; (**b**) *d* = 54 μm, *t* = 2.1 μm; (**c**) *d* = 71 μm, *t* = 2 μm; (**d**) *d* = 145 μm, *t* = 4 μm, respectively.

**Figure 9:**
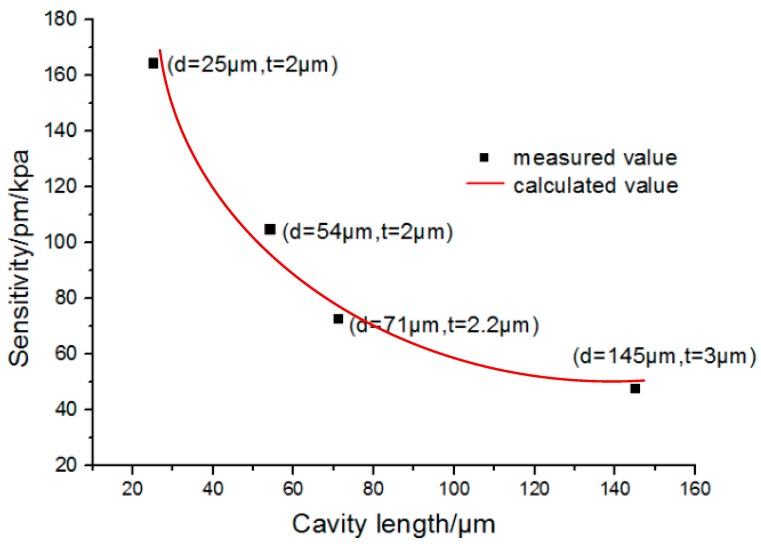
Relationship of the pressure sensitivity to the cavity length; the values (*d*, *t*) in brackets represent the air-cavity length and silica wall thickness.

To determine the relationship of the pressure sensitivity and the length of the fiber cavity, four similar microbubbles were used here, as shown in Figure 8. The wall thickness of the four microbubbles were around 2–4 μm. The maximum difference between those four microbubbles was that the cavity length was tuned between 254 and 1454 μm. It seems that with the increase of the cavity length, the corresponding calculated sensitivity vs. pressure were 164.56 pm/kPa, 105.08 pm/kPa, 71 pm/kPa, and 47.58 pm/kPa, respectively. Therefore, with the increase in the length of the cavity, the sensitivity decreases. Figure 9 shows a fitted curve of the sensitivity to cavity length. It can be determined from Figure 9 that with the increase of cavity length, the pressure sensitivity decreases significantly. While according to Equation (6), the sensitivity is inversely proportional to the cavity length. In the fitted line, the silica wall thickness is set as 2 μm. The result of this paper agrees with the theoretical model.

From the above results and Equation (6), it can be determined that the specific sensitivity is related to the thickness of the silica wall and the cavity length. As the single mode fiber was embedded into the microbubble in the second step, followed by the fabrication of microbubble, we were able to decrease the wall thickness of the microbubble in the first step and reduce the cavity length in the second step. A high-pressure sensitivity up to 164.56 pm/kPa was realized in our experiment. Compared with the results (1036 pm/MPa) of previous sub-micron silica diaphragm-based fiber-tip Fabry–Perot interferometers [10], a sensitivity enhancement of 158 times was realized in our experiment. Such progress can be attributed to the better controllability of the bubble thickness and cavity length.

Additionally, the fluctuation of temperature could also influence the application of the proposed fiber pressure sensor. For determining its temperature anti-interference ability, the influence of temperature fluctuations on the fabricated pressure sensor was also analyzed. The testing method entailed putting the pressure sensor in a temperature-controlled oven. The other end of the fiber pressure sensor was connected to the same interrogator to observe the reflected F-P interference spectrum. Then the oven temperature was increased gradually from 40 to 120 °C in increments of 20 °C. By measuring the shift of the reflected F-P interference spectrum, its temperature anti-interference ability could be determined.

**Figure 10:**
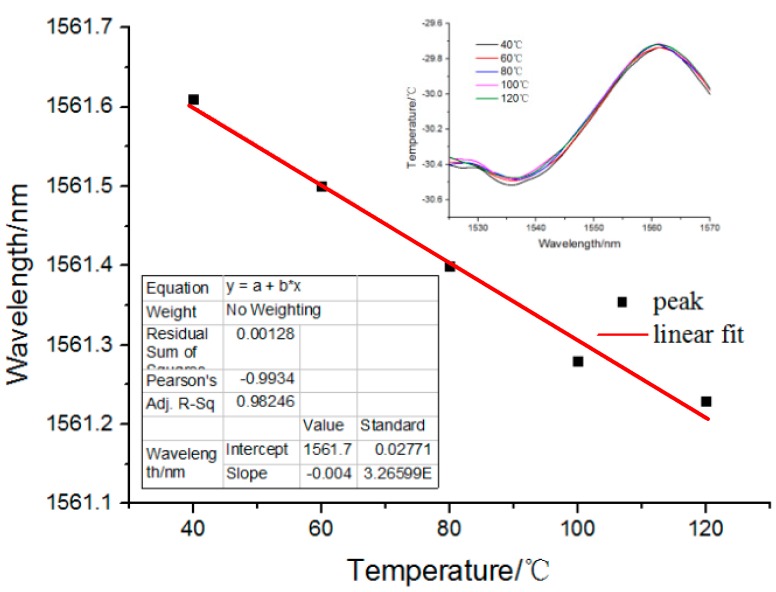
Temperature anti-interference ability of the fiber pressure sensor. Inset: the reflection spectra with the temperature ranging from 40 to 120 °C.

Figure 10 shows the wavelength shift of the fiber pressure sensor under different temperatures. The inset in Figure 10 shows that the reflection spectra shift with temperature ranging from 40 to 120 °C. For the reflected waves associated with the internal air-cavity, the thermally-induced refractive index change of air may be negligible [3] and the thermal expansion of the cavity length would then play a main role in the wavelength shift. Here Δλ/λ=ε+κ, where *ε* is the thermal expansion coefficient and κ is the thermo-optic coefficient. For pure silica, $\varepsilon =5.5\times 10^{-7}$, and $\kappa =1.0\times 10^{-5}$. As a result, we believe its temperature anti-interference ability was mainly due to the low thermal expansion of the silica wall, According to Equation (2), $\gamma =4\pi n_{air}d/\lambda$, the cavity length increases with the increase in temperature, which causes the wavelength shift. From the inset, we can observe that as the temperature increases, the reflection spectrum shifts slightly. The fabricated fiber pressure sensor shows a good temperature anti-interference ability in the measured temperature range. The temperature sensitivity was estimated to be 4 pm/°C. Thus, such a fiber sensor has the advantage of compact size, good mechanical strength, and high temperature stability. Additionally, it may be potentially used for pressure sensing in high-temperature environments, such as pipeline transmission and down-hole oil/gas exploration. The microbubble sensor at the end of the fiber has high sensitivity and low temperature sensitivity, which can obtain the pressure information in down-hole situations and eliminate the influence of the measured temperature field on the pressure detection.

## 4. Conclusions

In summary, a new fabrication method of a fiber pressure sensor based on the pressure-assisted arc discharge technique was presented. The wall thickness of the fabricated microbubble could be reduced to 2 μm. According to the reflection spectrum of this sensor under different pressures and temperatures, the pressure sensitivity is 164.56 pm/kPa and the temperature sensitivity is 4 pm/°C, which shows a good anti-interference ability. It has the advantage of low cost, compact size, good flexibility, easy fabrication, and low-pressure sensitivity. Such sensors may be used in many pressure measurement area, such as down-hole oil/gas exploration, which can obtain the pressure information while eliminating the influence of the temperature fluctuation.
